# Prediction of trust propensity from intrinsic brain morphology and functional connectome

**DOI:** 10.1002/hbm.25215

**Published:** 2020-10-01

**Authors:** Chunliang Feng, Zhiyuan Zhu, Zaixu Cui, Vadim Ushakov, Jean‐Claude Dreher, Wenbo Luo, Ruolei Gu, Xia Wu, Frank Krueger

**Affiliations:** ^1^ Key Laboratory of Brain, Cognition and Education Sciences (South China Normal University), Ministry of Education Guangzhou China; ^2^ School of Psychology, Center for Studies of Psychological Application, and Guangdong Key Laboratory of Mental Health and Cognitive Science, South China Normal University Guangzhou China; ^3^ School of Artificial Intelligence, Beijing Normal University Beijing China; ^4^ Engineering Research Center of Intelligent Technology and Educational Application of Ministry of Education, Beijing Normal University Beijing China; ^5^ Department of Psychiatry Perelman School of Medicine, University of Pennsylvania Philadelphia Pennsylvania USA; ^6^ National Research Center, Kurchatov Institute Moscow Russia; ^7^ National Research Nuclear University MEPhI, Moscow Engineering Physics Institute Moscow Russia; ^8^ Neuroeconomics, Reward and Decision Making Laboratory, Institut des Sciences Cognitives Marc Jeannerod, CNRS Bron France; ^9^ Research Center of Brain and Cognitive Neuroscience Liaoning Normal University Dalian China; ^10^ Key Laboratory of Behavioral Science, Institute of Psychology, Chinese Academy of Sciences Beijing China; ^11^ Department of Psychology University of Chinese Academy of Sciences Beijing China; ^12^ School of Systems Biology, George Mason University Fairfax Virginia USA; ^13^ Department of Psychology George Mason University Fairfax Virginia USA

**Keywords:** functional decoding, gray matter, individualized prediction, large‐scale brain networks, trust propensity

## Abstract

Trust forms the basis of virtually all interpersonal relationships. Although significant individual differences characterize trust, the driving neuropsychological signatures behind its heterogeneity remain obscure. Here, we applied a prediction framework in two independent samples of healthy participants to examine the relationship between trust propensity and multimodal brain measures. Our multivariate prediction analyses revealed that trust propensity was predicted by gray matter volume and node strength across multiple regions. The gray matter volume of identified regions further enabled the classification of individuals from an independent sample with the propensity to trust or distrust. Our modular and functional decoding analyses showed that the contributing regions were part of three large‐scale networks implicated in calculus‐based trust strategy, cost–benefit calculation, and trustworthiness inference. These findings do not only deepen our neuropsychological understanding of individual differences in trust propensity, but also provide potential biomarkers in predicting trust impairment in neuropsychiatric disorders.

## Graphical abstract

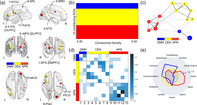

## 1 INTRODUCTION

Trust is an essential component of human relationships that is indispensable in interpersonal, institutional, and intercultural relationships (Fehr, 2009). Overall, trust refers to a person's (i.e., trustor) willingness to be vulnerable to the risk of betrayal based on the expectations that the action of another party (i.e., trustee) will produce some anticipated reward due to reciprocity in the future (Mayer, Davis, & Schoorman, 1995). As an important antecedent of trust behavior, propensity to trust is a trait‐based characteristic that refers to the general tendency for someone to trust others (Mayer et al., 1995). Evidence exists that propensity to trust has a global effect not only on trust intentions (Colquitt, Scott, & LePine, 2007) but also trustworthiness beliefs about others (Jones & Shah, 2016). The impact of trust propensity is most salient in early trustor‐trustee interactions, when other information about the trustee's trustworthiness may not be available (McKnight, Cummings, & Chervany, 1998). In the absence of other information about the trustee, the trustor's decision likely depends on an evaluation of the likelihood of incurring a loss versus being rewarded for trusting—thereby applying a calculus‐based trust strategy—based on the trustor's dispositional propensity to trust.

A widely‐used quantitative and reliable measurement of trust behavior is the sequential two‐person reciprocal trust game, for which the one‐shot version measures trust propensity toward an anonymous partner (Berg, Dickhaut, & McCabe, 1995; Camerer, 2003). Those tendencies to trust are relatively stable over the life course (Claibourn & Martin, 2000); however, individual differences exist: some people are almost completely willing to trust a stranger, whereas others display strong distrust. In this article, we investigated the neuropsychological mechanisms of the heterogeneity in trust propensity—employing the one‐shot version of the trust game—based on intrinsic structural and functional features of the brain.

Much of what we know about the neuropsychological mechanisms of trust is from task‐based functional magnetic resonance imaging (fMRI) studies that associate experimental conditions or behavioral performance with neural activation. Task‐based and meta‐analytic neuroimaging (Bellucci, Chernyak, Goodyear, Eickhoff, & Krueger, 2017; Bellucci, Feng, Camilleri, Eickhoff, & Krueger, 2018; Engelmann, Meyer, Ruff, & Fehr, 2019), lesion (Adolphs, Tranel, & Damasio, 1998; Belfi, Koscik, & Tranel, 2015), and oxytocin (Baumgartner, Heinrichs, Vonlanthen, Fischbacher, & Fehr, 2008; Nave, Camerer, & McCullough, 2015) studies have indicated trust as a complex psychological construct supported by multiple distributed regions. For example, the trust game consistently activates core regions, including subcortical (e.g., anterior insula, AI; amygdala, AMY; striatum, STR) as well as temporal (e.g., temporoparietal cortex, TPJ; temporal pole) and prefrontal (medial dorsomedial prefrontal cortex, DMPFC; ventromedial PFC, VMPFC; dorsolateral PFC, DLPFC; and ventrolateral PFC, VLPFC) cortical regions (Baumgartner et al., 2008; Tzieropoulos, 2013).

A recent neuropsychoeconomic model of trust proposes that trust arises through the interplay of psychological systems—motivation, affect, and cognition—that engage regions anchored in domain‐general large‐scale brain networks (Krueger & Meyer‐Lindenberg, 2019). Overall, a trustor faces an inherent social dilemma during the one‐shot trust game—measuring individual differences in trust propensity. The risk of treachery (affect, salience network, SAN: e.g., AI, AMY) contrasted with the anticipation of reward (motivation, reward network: e.g., STR, VMPFC) creates uncertainty. Two types of bounded rationality (cognition) can be employed to remove uncertainty—linked with the vulnerability of trusting another person. The SAN may engage the central‐executive network (CEN: e.g., DLPFC, VLPFC, posterior parietal cortex, PPC) adapting a context‐based strategy to reap personal benefits (i.e., economic rationality) and the default‐mode network (DMN: e.g., DMPFC, TPJ) evaluating the relationship‐based trustworthiness of a partner to contribute to the relationship's success (i.e., social rationality). Trust relationships evolve through different stages primarily driven by the DMN (evaluation of trustworthiness) and CEN (adoption of strategy): (a) from calculus‐based trust (i.e., trustors perform rational calculations of the costs and benefits of creating a relationship); (b) over knowledge‐based trust (i.e., trustors acquire additional knowledge about the contexts and their partners to predict trustees' behaviors accurately); (c) to identification‐based trust (i.e., trustors develop a rewarding identification and understanding with trustees to confidently trust them) (Lewicki & Bunker, 1995). Given the consistent involvement of these domain‐general large‐scale brain networks in trust, an intriguing question arises whether task‐independent structural and functional measures in these regions can predict individual differences in trust propensity.

Recent applications of task‐free brain morphology and resting‐state functional connectivity (RSFC) studies indicate that individual differences in intrinsic brain structures or functional connectome are closely related to individual differences in personality traits (Beaty et al., 2014; Dubois, Galdi, Han, Paul, & Adolphs, 2018; Jiao et al., 2017; Nostro et al., 2018) and social preferences (Baumgartner, Saulin, Hein, & Knoch, 2016; Campbell‐Meiklejohn et al., 2012; Feng et al., 2018). In terms of trust propensity, combining a self‐reported measure with voxel‐based morphometry (VBM) analysis showed an association with regional gray matter volume (GMV) changes in the DMPFC (Haas, Ishak, Anderson, & Filkowski, 2015). However, such type of univariate analyses only allows conclusion at the group level rather at the individual level; therefore, lacking out‐of‐sample generalizations that permit detecting complex brain‐behavior relationships (Dubois & Adolphs, 2016). Further, applying a multivariate predictive framework of individual differences in trust propensity can be predicted only for the one‐shot (measuring trust propensity) but not multi‐shot (measuring trust dynamics) trust game from RSFC relying on electroencephalography (EEG) activity over parietal regions (Hahn et al., 2014). Nevertheless, applying EEG‐based RSFC for source localization and drawing conclusions from importance scores of multivariate models are controversially discussed in the literature (Haufe et al., 2014). Finally, applying a machine learning approach, individual differences in trust propensity can be predicted from whole‐brain RSFC and RSFC from domain‐general large‐scale networks essential for the motivational, affective, and cognitive aspects of trust (Bellucci, Hahn, Deshpande, & Krueger, 2019; Lu et al., 2019). Such type of studies usually employs cross‐validation procedures to estimate the prediction model with training samples and to test the performance of the model with independent test samples**—**nonetheless without showing the generality of those predictions to an out of sample population performing another paradigm measuring trust propensity (Jung, Lee, Lerman, & Kable, 2018).

In this study, we extended those previous approaches—measuring mostly one modality focusing on RSFC—to overcome their limitations in two substantial aspects. We implemented an internal validation approach*—*applying a multivariate predictive framework (using machine learning)—to predict individuals' trust propensity based on two intrinsic brain features applying two task‐free neuroimaging modalities. Due to practical implications that brain morphometry can be more reliably collected than RSFC (Zuo, Xu, & Milham, 2019), we first predicted individual variations of trust propensity based on the regional GMV—a structural measure determined from structural MRI (sMRI). We next determined the node strengths of those identified anatomical regions within the same population—employing a graph‐theoretical measure of the centrality of a region computed from RSFC (Rubinov & Sporns, 2010)—to predict individual differences in trust propensity. Regions with high node strength (i.e., global FC strength, gFCS) have been regarded as functional hubs in domain‐general large‐scale brain networks (Buckner & Carroll, 2007; Wang, Dai, Gong, Zhou, & He, 2015).

Moreover, we implemented a trust prediction model based on the intrinsic structural brain features in two independent samples that performed two different versions of the one‐shot trust game. The first sample (undergoing the internal validation) completed the standard trust game, whereas the second sample (undergoing the external validation) completed the binary trust game. Finally, we performed a modular analysis (i.e., community detection algorithm) to detect network connectivity patterns (i.e., modules) among the identified anatomical regions as well as a functional decoding analysis to link the identified modules with psychological functions. These following‐up analyses aimed to provide data‐driven quantitative inference on psychophysiological functions of contributing regions.

Based on the neuropsychoeconomic model of trust (Krueger & Meyer‐Lindenberg, 2019), we assumed that participants completing the one‐shot trust game with an anonymous partner would apply a calculus‐based trust strategy to remove uncertainty; therefore, transforming the risk of treachery into positive expectations of reciprocity. As a measure of individual differences in trust propensity, calculus‐based trust engages both the DMN to simulate the trustworthiness of the partner and the CEN to apply rational costs‐benefits calculations. Hence, we hypothesized that intrinsic structural (GMV) and functional (gFCS) features of regions being part of DMN and CEN predict individual differences in trust propensity. Combining an MVPA framework with modular and functional decoding analyses, our findings demonstrated that individual differences in trust propensity are decoded by intrinsic structural and functional characteristics of modules being part of DMN and CEN in addition to an action‐perception network (APN) module associated with number processing—as measured in two different populations and trust paradigms.

## 2 METHODS

### 2.1 Participants

The current study consisted of two independent samples of healthy right‐handed participants without a history of neurological or psychiatric disorders, playing two different versions of the one‐shot trust game. The first sample (playing the standard version of the trust game) included 89 college students (45 males; 26 [mean] ± 2.22 [*SD*] years old, range: 18–27 years old) and the second sample (playing a binary version of the trust game) included 86 college students (73 males; 22.62 ± 2.37 years old, range: 18–30 years old). Although both samples were collected from the same site at Beijing Normal University; however, they were recruited in different projects. Participants gave written informed consent for this study, which was approved by the Ethics Committee of Beijing Normal University and conducted following the Declaration of Helsinki.

### 2.2 Economic games

The first sample played a one‐shot dictator game as dictators (Kahneman, Knetsch, & Thaler, 1986) and a one‐shot trust game as trustors (Berg et al., 1995) with different anonymous partners. Participants were given written instructions about both games and asked to answer several questions to assess their understanding. In the dictator game, participants decided how to split a sum of money (12 monetary units, MUs) between themselves and the other player as a passive recipient. People's behaviors in the dictator game have been argued to reflect generosity or altruistic preferences (Benenson, Pascoe, & Radmore, 2007). In the trust game, participants started with an endowment of nine MUs and decided whether to trust or not by sending any portion of the endowment to the trustee and to keep the remainder of the endowment. The shared money was tripled in value by the experimenter and passed on to an anonymous partner, who would participate as a trustee in another experiment to decide how much to return. The first sample was reported in our recent study employing an independent prediction scheme (Lu et al., 2019).

The second sample played a one‐shot dictator game as dictators and one‐shot binary trust game as trustors. In the dictator game, participants decided how to allocate 100 MUs between themselves and the other player as a passive recipient. The one‐shot binary trust game was similar to the standard game, except that trustors faced a binary choice to either keep or give all of their endowment to the trustees. According to their choices (i.e., trust or distrust), participants were categorized into a trusting (*n* = 35) or distrusting (*n* = 51) group. The two groups did not differ in gender, age, and brain size (All *p* > .05, Table S1). Participants in the second sample did not play standard trust game as played by the participants in the first sample, since these two datasets were collected in different projects rather than being designed as a single study.

Participants came to the lab only once to complete the study. They were informed that they would be paid a week later after another group of participants completed their decisions as trustees in the trust game. Unknown to the first group of participants, no trustees were recruited for the experiment; however, to encourage real decisions, it was emphasized that the earned MUs from the games would be converted into the monetary payout. Since the exact exchange rate (from MUs to monetary payouts) was unknown, participants were all paid with a fixed amount (50 RMB) about a week later. Before leaving the laboratory, participants were debriefed to examine their beliefs about the experimental setup and none of them expressed doubts about the implemented procedure.

### 2.3 Image acquisition

MRI acquisition was performed with a Siemens Trio 3‐Tesla scanner at the Beijing Normal University Imaging Center for Brain Research. High‐resolution structural images were acquired through a 3D sagittal T1‐weighted magnetization‐prepared rapid acquisition with gradient‐echo (MPRAGE) sequence, using the following parameters: sagittal slices, 144; repetition time (TR), 2,530 ms; echo time (TE), 3.39 ms; slice thickness, 1.33 mm; voxel size, 1 × 1 × 1.33 mm^3^; flip angle, 7°; inversion time, 1,100 ms; and field of view (FOV), 256 × 256 mm^2^.

The qualities of all T1‐weighted images were good or satisfactory, and no participants were excluded (Supporting Information, Section 1, Figure S1). The first sample also completed a 5‐min resting‐state fMRI (rs‐fMRI) scan consisted of 150 contiguous echo‐planar imaging (EPI) volumes using the following parameters: axial slices, 33; slice thickness, 3.5 mm; gap, 0.7 mm; TR, 2,000 ms; TE, 30 ms; flip angle, 90°; voxel size, 3.5 × 3.5 × 3.5 mm^3^; and FOV, 244 × 244 mm^2^. During the RS scan, participants were instructed to close their eyes, keep still, remain awake, and not to think about anything systematically. Several approaches were implemented to prevent the participants from falling asleep during the scan. They were explicitly instructed to close their eyes but not fall asleep during the scan. Experimenters communicated with each participant immediately after the scan, and they all responded promptly, indicating that they did not fall asleep.

### 2.4 Gray matter volume (GMV) features

From each participant, a GMV map was obtained in the Montreal Neurological Institute (MNI) space using VBM8 implemented with Statistical Parametric Mapping (SPM8; Wellcome Trust Centre for Neuroimaging, http://www.fil.ion.ucl.ac.uk/spm/) on a MATLAB (MathWorks, Natick, MA) platform. The processing procedure consisted of the following steps. First, the original T1‐weighted image of each participant was reoriented to the center point of the anterior commissure. Second, volumetric T1‐weighted images were segmented into GM, white matter, and cerebrospinal fluid density maps using the standard unified segmentation approach (Ashburner & Friston, 2005). Third, the segmented GM density (GMD) map was spatially normalized to the International Consortium for Brain Mapping (ICBM) GM template. Fourth, the modulation was applied to the normalized GMD images by multiplying the nonlinear components of Jacobian determinants, resulting in GMV maps adjusting for individual variations in brain sizes. Fifth, the resultant GMV maps were smoothed using a 4‐mm full‐width at‐half‐maximum (FWHM) Gaussian kernel. Finally, to create a GM mask, each GMD map was also smoothed with a 4‐mm FWHM kernel size. Those GMD maps were then averaged, and a threshold of 0.2 was applied to this average map (Cui, Su, Li, Shu, & Gong, 2018). The GMV features were restricted to the GM mask to maintain the consistency of the feature dimensions across all participants (Köbe et al., 2016).

### 2.5 Multivariate prediction model

For the standard trust game completed by the first sample, an elastic‐net regularized linear regression model was applied to predict trust propensity at the individual level. This regularized linear prediction model has been successfully applied in previous studies (Cui et al., 2018; Khundrakpam, Tohka, & Evans, 2015). Voxel‐wise GMV features in a linear regression model were formalized as follows:

$$y=\sum_{i=1}^{p}\beta_{i}x_{i}+\beta_{0}$$

where *y* is the individual's trust preference, *p* is the number of voxels in the GM mask, *x*_*i*_ is the GMV value at the *i*th voxel, and *β*_*i*_ is the corresponding regression coefficient.

To alleviate the issues of multicollinearity and overfitting, regularization methods have been frequently applied (Friedman, Hastie, & Tibshirani, 2010). The L1‐norm regularization—least absolute shrinkage and selection operator (LASSO)—minimizes the sum of the absolute regression coefficients and keeps only one representative predictor from the correlated predictors. The LASSO achieves a sparse model by excluding the majority of features from the model; therefore, facilitating the optimization of the predictors and reducing the model complexity. However, the LASSO can only retain *N* (i.e., sample size) features at the most in the final model. This is problematic for a model with relatively small samples but a much larger number of features such as in our study that includes 89 samples but more than 180,000 features.

In contrast, the L2‐norm regularization (i.e., ridge regression) minimizes the sum of the square of the regression coefficients and keeps all features in the model. Finally, elastic‐net regularization reflects a weighted combination of L1‐norm and L2‐norm regularization and retains the desirable property of LASSO by providing sparse solutions while overcoming the problems associated with a large number of voxel features. Specifically, the elastic‐net regularization takes the following form, which was added to the linear regressions model as a penalty term:

$$\lambda \sum_{j=1}^{p}\left(\alpha {\left\|\beta_{j}\right\|}_{L1}+\frac{1}{2}\left(1-\alpha \right){\left\|\beta_{j}\right\|}_{L2}^{2}\right)$$

where *β*_*j*_ is the regression coefficient for the *j*th feature, and *α* is a mixing parameter that controls the relative weighting of the L1‐norm and L2‐norm contributions. The regularization parameter *λ* controls the amount of shrinkage that was applied to *β*_*j*_. If *λ* = 0, the effect of the elastic‐net penalty is canceled. As *λ* increases from zero, the coefficients are progressively shrunk. Elastic‐net uses *α* to create a useful trade‐off between ridge and LASSO, which is equivalent to the ridge regression when *α* = 0 and is equivalent to the LASSO when *α* = 1. The present study applied the scikit‐learn library (version: 0.19.1) to implement the elastic‐net regularization regression (http://scikit-learn.org/).

### 2.6 Multivariate prediction framework

**FIGURE 1:**
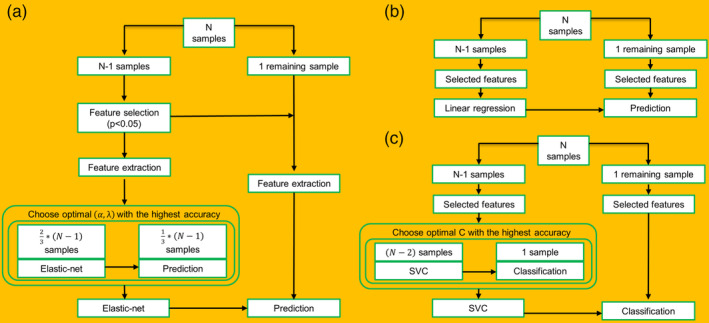
Prediction framework. (a) The prediction schematic flow using GMV‐based features and elastic‐net. (b) Internal validation using node strength‐based features of selected voxels. (c) External validation using GMV‐based features of selected voxels. SVC, support vector classifier

For the prediction framework, nested cross‐validation was adopted with an outer leave‐one‐out cross‐validation (LOOCV) to evaluate the prediction performance and inner three‐fold cross‐validation used to select the optimal parameters **(**Figure 1a). First, the GMV values of all GM voxels in the GM mask were extracted to generate the feature vector for each participant. Second, a feature‐selection scheme was implemented, such that the correlation between each feature and trust propensity was computed within the training sample on each iteration of LOOCV. The resultant correlation coefficients were forward to a threshold of uncorrected *p* < .05 (Xie et al., 2015). The selected features (i.e., voxels) were applied to the remaining GMV map of the testing participant. Third, a grid search was conducted to determine the optimal parameter (*α*, *λ*) set for the elastic‐net regularization model.

For the outer loop, supposing the whole dataset consisted of *N* participants, then *N* − 1 participants were used for the feature selection and training for an optimal prediction model. The remaining participant was used for testing to evaluate the prediction accuracy of the model. The procedure was repeated *N* times, each time leaving out a different participant for testing, resulting in *N* predictions, and one for each participant. Estimates such as Pearson's *r* and *mean squared error* (*MSE*) between actual and predicted trust propensity were used to evaluate the accuracy of prediction. Within each outer loop, the optimal parameter set for the elastic‐net regularization model was determined using cross‐validations.

For the loop of the inner cross‐validation, the training set (*N* − 1) was further partitioned into three subsets according to their rank of the behavioral scores (Callie, Palma, Charles, & Ariun, 2013). Each inner loop procedure was then executed under a given parameter set of (*α*, *λ*). Regarding the (*α*, *λ*) choices, a grid search was employed: the *α* was chosen from 10 values in the range of [0.2, 1.0] and the *λ* was set as *λ* = *e* ^*γ*^, where *γ* was chosen from 20 values in the range of [−6, 5] (Barretina et al., 2012). This scheme resulted in a total of 200 (*α*, *λ*) parameter sets. For each set of (*α*, *λ*), Pearson's *r* and *MSE* between the predicted trust propensity and the actual trust propensity were computed to quantify the accuracy of the prediction. Then, the (*α*, *λ*) set with the highest inner prediction accuracy across the 200 inner three‐fold CV loops was chosen as the optimal parameter set for the final elastic‐net predictive model, which was applied to the training set of the corresponding outer LOOCV loop (Cui et al., 2018).

### 2.7 Significance of prediction performance

To determine whether the obtained final prediction results (i.e., Pearson's *r* or *MSE*) were significantly better than expected by chance (Dosenbach & Schlaggar, 2010), a permutation test was applied to permute trust propensity 1,000 times randomly. For each time, the above prediction procedure was executed repeatedly only with randomly permuted trust propensity. The *p*‐value of the Pearson's *r* was calculated by dividing the number of permutations that showed a higher value than the actual value for the real sample by the total number of permutations (i.e., 1,000). Similarly, the *p*‐value of the *MSE* was the portion of permutations that showed a lower value than the actual value for the real sample.

### 2.8 Contributing GM voxels

After the training procedure of the model was accomplished, the features (i.e., GM voxels) with a nonzero regression coefficient/weight in the models of all outer LOOCV loops can be defined as the contributing voxels for the prediction of trust propensity (Khundrakpam et al., 2015). The absolute regression coefficient/weight of a voxel represents the importance of the GMV feature in predicting trust propensity (Cui et al., 2018; Dosenbach & Schlaggar, 2010). The intersection of features with nonzero regression coefficients across all outer LOOCV loops was selected as brain measures predictive of trust propensity.

Note that a control analysis was implemented to examine further the significance of predictions for the model—controlling for potential confounds of altruistic preferences, age, gender, individual brain size, and T1‐weighted image quality rating. In particular, the association between actual and predicted trust propensity was re‐computed based on the residuals after adjusting for these confounding variables.

### 2.9 External validation: Classification of trust propensity in an independent sample

As a test of generalizability, the identified GMV features were employed to an independent validation sample of participants who played the binary trust game. GMV information with the selected contributing voxels in the new dataset was applied to classify trusting and distrusting groups. A linear support vector classification (LSVC) algorithm implemented in MATLAB (MathWorks, Natick, MA) and LIBSVM toolbox (http://www.csie.ntu.edu.tw/~cjlin/libsvm/) was used. The LSVC allows for finding optimal weights and bias for a discriminant function using a training data set, which is identified as a hyperplane in this multidimensional space to best separate the training data into two categories matching with the known labels (i.e., trusting or distrusting group). The discriminant function was, in turn, applied to predict the class of new testing samples by the output of classification scores (positive scores indicate a trusting group, and negative scores indicate a distrusting group). The LSVC was employed because (a) the predictive regression model trained on the first dataset cannot be directly used for the second dataset concerning the classification problem and (b) the LSVC is one of the most widely used supervised classification algorithms in the field of neuroscience and usually outperforms other methods (Misaki, Kim, Bandettini, & Kriegeskorte, 2010).

A nested cross‐validation scheme was implemented to train the model and optimize the hyperparameter of the model (soft‐margin parameter, C), with the outer loop for examining the performance of the model and the inner loop for optimizing the hyperparameter in the range of [0.005, 0.01, 0.02, 0.1, 0.2, 0.5, 1, 2, 5, 10, 50, 100, 200, 1,000, 2000, 5,000] (Figure 1c). The LOOCV was adopted for both inner and outer loops (Feng et al., 2018). To quantify the classification performance, accuracy, sensitivity, specificity, positive predictive value (PPV), and negative predictive value (NPV) were calculated. Accuracy refers to the proportion of participants who were correctly classified into the trusting or distrusting group. Sensitivity and specificity are the proportion of trusting group and distrusting group classified correctly, respectively. PPV and NPV are the proportion of correct trusting group and distrusting group predictions, respectively. Also, a receiver operating characteristic (ROC) analysis was implemented to evaluate the performance of the model. The area under the curve (AUC)‐ROC represents the classification power of a classifier, such that a larger AUC indicates a higher classification power. The ROC curve was generated with sequential thresholding at the classification score of each participant.

Finally, the significance of the classification accuracy and AUC was determined using a permutation test with 1,000 permutations. The algorithm was fitted to randomly permuted targets using the above‐described LOOCV procedures for a total of 1,000 permutations. In every permutation, the LOOCV approach was employed to fit the classification model to randomly permuted targets. The *p‐*value for the classification accuracy was calculated by dividing the number of models with randomly permuted targets that showed higher accuracy than that of the model with true targets by the total number of permutation (i.e., 1,000).

### 2.10 Ten‐fold validation

Each of the above prediction or classification models was validated with ten‐fold cross‐validation, which might provide more stable estimates of predictive accuracy than the LOOCV scheme (Varoquaux et al., 2017). Participants were divided into 10 subsets, of which nine were used as the training sets, and the remaining one was employed as the testing set. This procedure was repeated 10 times so that each subset was used as a testing set once. Since the full dataset was randomly divided into 10 subsets, performance might have depended on data division. Therefore, the ten‐fold cross‐validation was repeated 100 times, and the results averaged to produce a final prediction performance. A permutation test was applied 1,000 times to test the significance of the prediction performance.

### 2.11 Internal validation: Prediction from RSFC features of selected voxels

Employing voxels identified in the GMV‐based predictive model, it was next examined whether the prediction model generalized to the RSFC‐related measures of these voxels. A similar prediction framework was implemented except that (a) the feature selection or the elastic‐net regularization scheme was not employed, that is, all the voxels contributing to the GMV‐based prediction model were included (Figure 1b) and (b) gFCS rather than GMV values on each voxel was employed as predictors. A linear regression model combining with a cross‐validation scheme was employed in the prediction of trust propensity based on gFCS features. The internal validation based on functional characteristics was inspired by a growing body of evidence that brain structure characteristics (e.g., gray matter volume) have a direct impact on brain function, causing functional differences (Honey, Thivierge, & Sporns, 2010; Neudorf, Ekstrand, Kress, & Borowsky, 2020; Tewarie et al., 2014). Therefore, the identified contributing structural voxels are also most likely to be the functionally affected ones related to human's corresponding cognition and behavior. Likewise, a plethora of evidence shows that abnormal brain structure and corresponding changes in brain function can strongly affect behavior (Achiron et al., 2013; Schneider et al., 2005; von dem Hagen et al., 2011), further illustrating the necessity of the corresponding functional features in behavior prediction. Hence, we examined whether these identified structural voxels would be functionally predictable.

The voxel‐wise node strength analysis was performed using the GRETNA (GRaph thEoreTical Network Analysis) toolbox (http://www.nitrc.org/projects/gretna/) (Wang, Wang, et al., 2015). A whole‐brain voxel‐wise functional connectivity (FC) matrix for each participant was obtained by computing the correlation (Pearson's *r*) between the time series of each pair of brain voxels in the brain mask and then converted to the Fisher's *Z*‐values (Figure S3). For a given voxel, its node strength value was defined as the sum of the *Z*‐values between the voxel and all of the other voxels in the brain mask. As such, node strength characterizes the degree of centrality for a given brain region without referring to its functional connectivity with a particular area (Wang, Wang, et al., 2015). As voxel‐wise GMV, gFCS is also a nodal feature; therefore, providing a comparable feature derived from functional data. Specifically, the node strength approach does not require selection of a priori nodes or networks of interest, thus allowing for a comprehensive, whole‐brain characterization for the FC property of each voxel across the whole brain (Gotts et al., 2012). Moreover, node strength has been employed as a RSFC measure in numerous studies (Cao et al., 2018; Feng et al., 2018; Jung et al., 2018; Wu et al., 2015) and brain regions with high node strength have been regarded as functional hubs in large‐scale brain networks (Buckner & Carroll, 2007; Wang, Dai, Gong, Zhou, & He, 2014).

A control analysis was implemented to further examine the significance of the predictions of the model after controlling for potential confounds of altruistic preferences, age, gender, head motion, and T1‐weighted image quality rating. In detail, after adjusting for these confounding variables, the association between actual and predicted trust propensity was re‐computed. Finally, we implemented an additional control analysis to examine the prediction of trust propensity based on whole‐brain gFCS features. The prediction framework was the same as the main model except that the prediction features were gFCS measures rather than GMV.

### 2.12 Modular analysis of the functional brain network

A modular analysis of the functional brain network was performed to explore the potential functionally specific relationships between these contributing clusters. Regions of interest (ROIs, *n* = 13) were defined by building spheres with a 4 mm radius around the contributing cluster peak voxels. The mean time courses of all the voxels within each ROI were extracted to calculate the Pearson correlation coefficient matrix for representing the resting brain functional network, resulting in a symmetric connectivity matrix for each participant. These matrices were Fisher *z*‐transformed and averaged to obtain a mean matrix used for the following analyses.

To exclude the confounding impact of spurious relationships in internal connectivity matrices, the obtained mean matrix connectivity density value was set to range from 0.26 to 0.50 with a step length of 0.01. These low‐value filtered matrices were performed for the modular analysis using the Graph‐Theoretical Network Analysis Toolkit (Wang, Wang, et al., 2015). The toolkit detects communities by maximizing the modularity Q with the spectral optimization algorithm, which has been introduced as a measure to assess the goodness of a partition (Newman, 2006; Newman & Girvan, 2004). Finally, the number of modules and the membership of each ROI were obtained.

### 2.13 Functional decoding for contributing modules

To explore which psychological topics were most relevant to the three identified modules, a meta‐analysis was first performed based on version 0.6 of the Neurosynth database (Yarkoni, Poldrack, Nichols, Van Essen, & Wager, 2011). The database consists of 11,406 fMRI studies and over 410,000 activity peaks that cover all‐sided published neuroimaging literature (Figure S2). The observations for each study contains the peak activities for all contrasts reported in the study's table and the frequency of all words in the article abstract. Notably, a set of psychological 60 topics were used (De La Vega, Yarkoni, Wager, & Banich, 2017), which was derived by the latent Dirichlet allocation topic modeling to remedy the redundancy and potential ambiguity in word terms (Blei, Ng, & Jordan, 2003).

Using all fMRI studies, next, a functional decoding analysis was performed by training a naïve Bayes classifier, which is widely used in text classification (Lewis, 1998; Rennie, Shih, Teevan, & Karger, 2003). Two sets of studies that activated at least 5% voxels and that did not activate any voxel of the given ROI were selected respectively, as the positive and negative samples of the training set (De La Vega et al., 2017). The AUC‐ROC was used to measure the performance of the model with a four‐fold cross‐validation. This resulted in the conditional probability of the 60 psychological topics under each module. Notably, only those topics that survived multiple comparisons using false discovery rate (FDR) with *p* < .01 by implementing a permutation test were reported. Finally, the log odds ratio between the probability of a given topic activating the module and the probability of the topic not activating the module was extracted from the trained naïve Bayes model to generate functional decoding profiles.

## 3 RESULTS

### 3.1 Performance of GMV‐based prediction model

**FIGURE 2:**
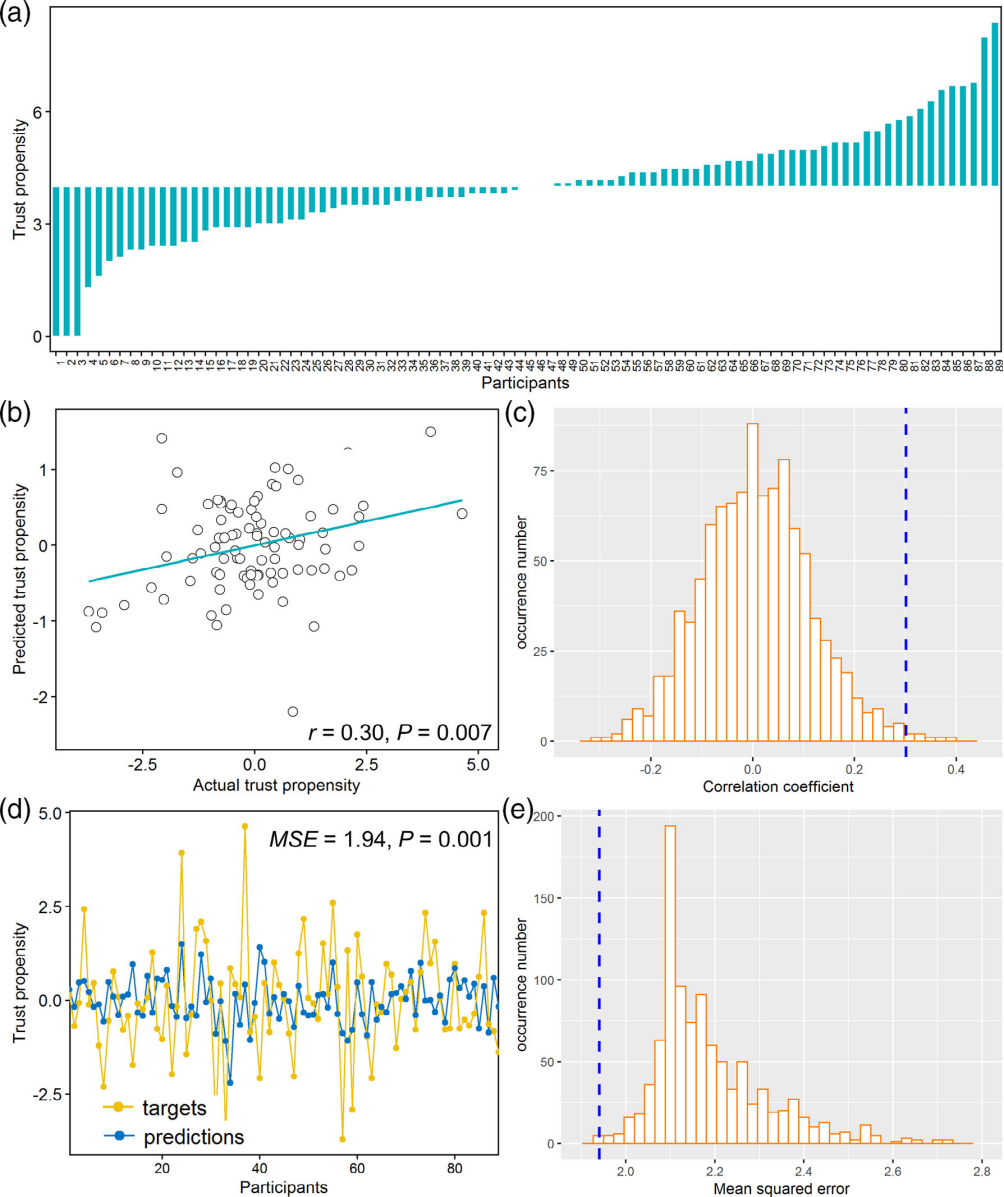
Performance of the GMV‐based prediction model. (a) Trust propensity (i.e., amounts of investment in the standard trust game: mean ± *SEM*: 4.00 ± 0.16) across participants. (b) Correlation between actual and predicted trust propensity. (c) Permutation distribution of the correlation coefficient (*r*) with blue dashed line indicating value obtained from real scores. (d) Consistency between actual and predicted trust propensity. (e) Permutation distribution of the mean squared error with blue dashed line indicating value obtained from real scores

First, we aimed to predict individual differences in trust propensity measured with the standard trust game (Figure 2a), combining an elastic‐net regularized linear regression GMV‐based model with a LOOCV approach.

The GMV features predicted individual differences in trust propensity (*r* = 0.33, *p* = .004; *MSE* = 2.06, *p* < .001, permutation test) and remained significant after adjusting for covariates such as altruistic preferences, age, gender, brain size, and image quality (*r* = 0.30, *p* = .007, Figure 2b,c; *MSE* = 1.94, *p* = .001, Figure 2d,e, permutation test). The reliability and significant of the model were further confirmed after performing a ten‐fold cross‐validation (unadjusted for covariates: *r* = 0.32, *p* < .001; *MSE* = 2.08, *p* < .001, permutation test; adjusted for covariates: *r* = 0.29, *p* = .007, Figure S3a,b; *MSE* = 1.96, *p* = 0.008, Figure S3c,d, permutation test).

### 3.2 Contributing regions of the GMV‐based prediction model

**FIGURE 3:**
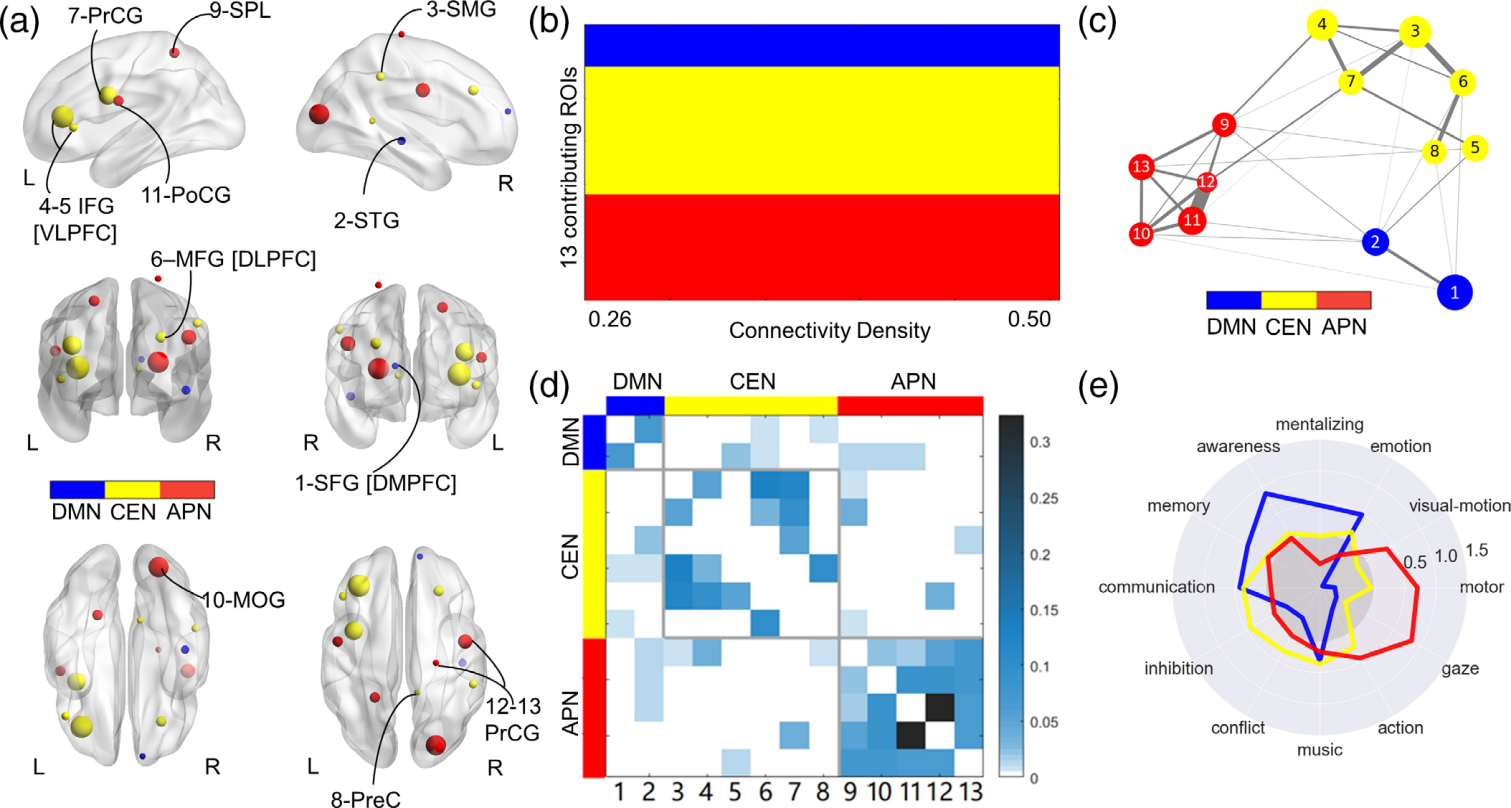
Contributing regions of the GMV‐based prediction model. (a) The GMV‐based prediction model determined 13 contributing regions (i.e., region of interests, ROIs) plotted with cluster sizes as the number of voxels. The colors indicate different brain network modules. (b) The modular analysis determined three stable modules from ROIs shown in the same color (default‐mode network, DMN, blue; central‐executive network, CEN, yellow; and action‐perception network, APN, red) under connectivity density levels ranging from 0.26 to 0.50 by increments of 0.01. (c) The spring‐like layout of the three network modules for a connectivity density of 0.40 displays the Euclidean distance between each pair of nodes, reflecting the graph‐theoretic distance and the thickness of lines, reflecting the connection strength of the edges. (d) Functional connectivity matrix for a connectivity density of 0.40 (ROIs are sorted by modules) showing a stronger strength of edges within than those between modules. (e) The log odds ratio displaying the functional decoding profiles for the top four psychological topics associated with each module. IFG, inferior frontal gyrus (ventrolateral prefrontal cortex, VLPFC); MFG, middle frontal gyrus (dorsolateral prefrontal cortex, DLPFC); MOG, middle occipital gyrus; PrCG, precentral gyrus; PoCG, postcentral gyrus; PreC, precuneus; SFG, superior frontal gyrus (dorsomedial prefrontal cortex, DMPFC); SMG; supramarginal gyrus; SPL, superior parietal lobule; STG, superior temporal gyrus

Next, we determined the representative voxels selected by the prediction model as important features in predicting trust propensity. Contributing voxel clusters were located in the following regions: superior temporal gyrus (STG, Brodmann area, BA 22), supramarginal gyrus (SMG, BA 40), superior parietal lobule (SPL, BA 7), precentral gyrus (PrCG, BA 9 & BA 6), postcentral gyrus (PoCG, BA 4), superior frontal gyrus (SFG, BA 10, DMPFC), inferior frontal gyrus (IFG, BA 45 & BA 46, VLPFC), middle frontal gyrus (MFG, BA 9, DLPFC), precuneus (PreC, BA 29) and middle occipital gyrus (MOG, BA 18) (Table 1, Figure 3a).

**TABLE 1:** Contributing regions in the GMV‐based prediction model

Region	BA	ROI ID	Hemi	Cluster size (voxels)	Peak MNI coordinate			Weights	Module
					*x*	*y*	*z*		
Superior frontal gyrus (SFG)	10	1	R	5	12	58	16	12.38	1
Superior temporal gyrus (STG)	22	2	R	6	44	−22	−6	7.07	1
Supramarginal gyrus (SMG)	40	3	R	6	52	−38	42	10.18	2
Inferior fontal gyrus (IFG)	46	4	L	13	−34	36	10	9.53	2
Inferior fontal gyrus (IFG)	45	5	L	6	−46	28	2	6.92	2
Middle frontal gyrus (MFG)	9	6	R	7	26	32	32	6.89	2
Precentral gyrus (PrCG)	9	7	L	11	−38	2	26	6.27	2
Precuneus (PreC)	29	8	R	5	10	−44	8	5.76	2
Superior parietal lobule (SPL)	7	9	L	7	−22	−48	58	5.67	3
Middle occipital gyrus (MOG)	18	10	R	12	24	−84	14	5.76	3
Postcentral gyrus (PoCG)	4	11	L	7	−50	−6	22	7.84	3
Precentral gyrus (PrCG)	6	12	R	9	46	−6	32	3.88	3
Precentral gyrus (PrCG)	6	13	R	5	24	−22	74	6.62	3

Abbreviations: BA, Brodmann area; Hemi, hemisphere; L, left; ROI, region of interest; R, right.

### 3.3 Internal validation: Prediction from RSFC features of selected voxels

**FIGURE 4:**
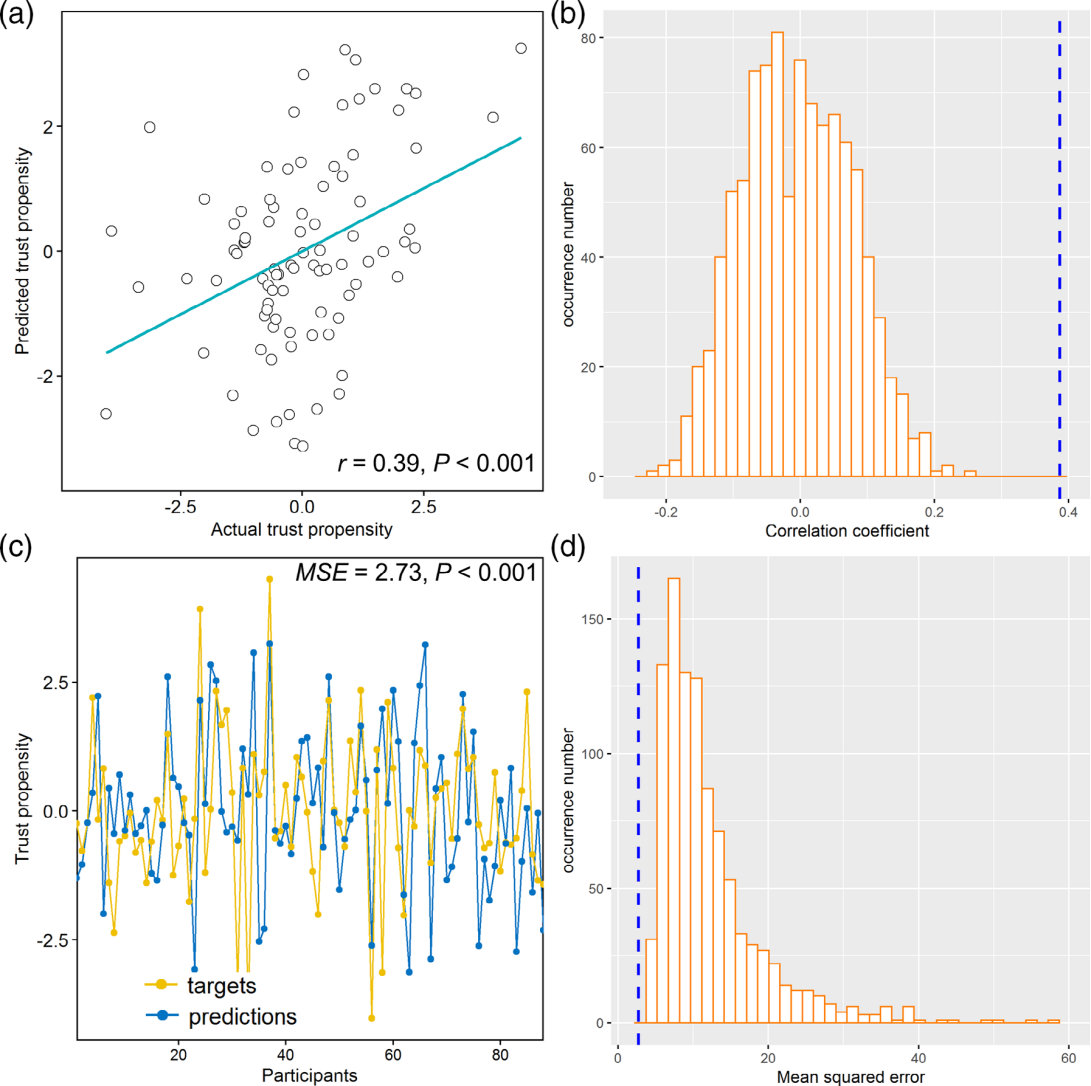
Internal validation of prediction model using node strength‐based features from selected voxels. (a) Correlation between actual and predicted trust propensity. (b) Permutation distribution of the correlation coefficient (*r*) with blue dashed line indicating value obtained from real scores. (c) Consistency between actual and predicted trust propensity. (d) Permutation distribution of the mean squared error with blue dashed line indicating value obtained from real scores

Next, we tested whether individual trust propensity can be predicted by node strength features (i.e., gFCS) of brain systems—a graph‐theoretical measure of the centrality of a region computed from RSFC (Rubinov & Sporns, 2010)—identified by the GMV‐based prediction model. The node strength of selected voxels for those regions were able to predict trust propensity (*r* = 0.39, *p* < .001; *MSE* = 2.89, *p* < .001, permutation test). The prediction remained significant after adjusting for altruistic preferences, age, gender, and head motion (*r* = 0.39, *p* < .001, Figure 4a,b; *MSE* = 2.73, *p* < .001, Figure 4c,d, permutation test) (Table S2). In contrast, the prediction model based on the whole‐brain gFCS features cannot reliably predict trust propensity (Supporting Information, Section 2).

### 3.4 External validation: Classification of trusting and distrusting groups

**FIGURE 5:**
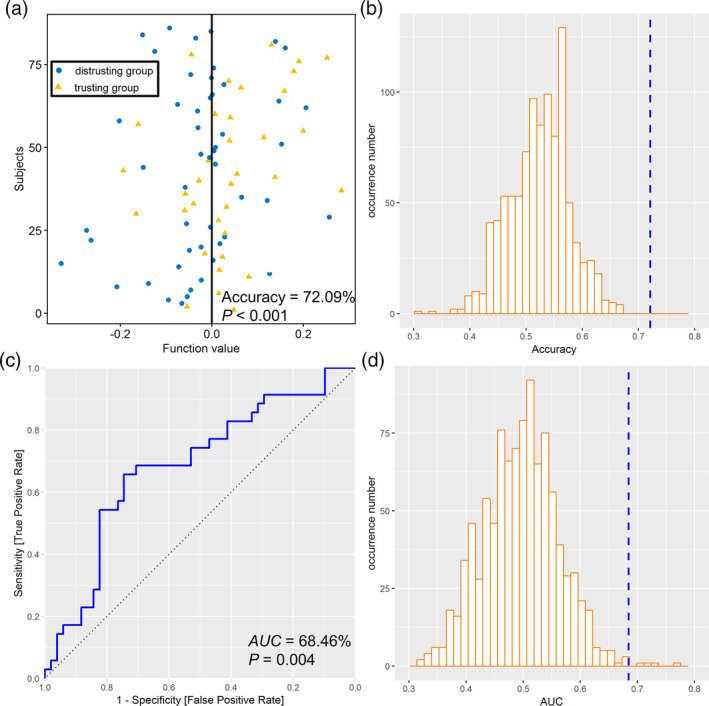
External validation based on GMV features from selected voxels. (a) The classification plot for each participant based on GMV features. (b) Permutation distribution of the classification accuracy with blue dashed line indicating the value obtained from real scores. (c) The receiver operating characteristic (ROC) graph for the GMV feature‐based classifier. (d) Permutation distribution of the area under the curve (AUC) with blue dashed line indicating the value obtained from real scores

Moreover, we performed an external validation using a new dataset (a second sample that completed the binary trust game) to classify trusting and distrusting groups—employing only voxels identified by the prediction model from the first sample. The LSVC (Table S3) accurately discriminated the two groups (accuracy, 72.09%; AUC, 68.46%; sensitivity, 76.47%; specificity, 65.71%; PPV, 76.47%; NPV, 65.71%). The permutation tests yielded *p* < .001 for accuracy (Figure 5a,b) and *p* = .004 for AUC (Figure 5c,d). Importantly, the GMV‐based model did not predict participants' altruistic preferences as measured with the dictator game, although trusting and distrusting groups exhibited significant differences in altruistic preferences (Supporting Information 3).

### 3.5 Network analysis for contributing regions

Furthermore, we performed a modular analysis (i.e., a community detection algorithm) to detect the connectivity patterns between the identified contributing regions. Three stable network modules were detected—DMN (blue), CEN (yellow), and APN (red)—for which the modules' partitioning maintained good consistency across different connectivity strengths (Figure 3b). For the connectivity density of 0.40, a spring embedder layout model for straight‐line representations was applied—grouping together or pulling apart nodes according to their connectivity patterns (Brandes & Wagner, 1997). The spring‐like layout of the three network modules was characterized by the Euclidean distance between each pair of nodes (reflecting the graph‐theoretic distance) and the thickness of lines (representing the connection strength of the edges) (Figure 3c). The RSFC for which ROIs were sorted by modules demonstrated that the modules were more strongly connected internally than externally (Figure 3d).

### 3.6 Functional decoding for contributing modules

Finally, we explored the modules' psychological functions by employing a data‐driven approach to survey a broad range of fMRI studies in the Neurosynth database (Yarkoni et al., 2011). Naïve Bayes classifiers were trained to predict the presence or absence of activity in each contributing module using a set of 60 psychological topics derived from a standard topic modeling approach to the article abstracts of the Neurosynth database. After training the model for each psychological topic, each topic received a conditional probability coefficient for activating a contributing module. The log odds ratio between the probability of a given topic activating the module and the probability of the topic not activating the module was displayed in a functional decoding profile for the top four topics per module (Figure 3e). Values greater than zero indicate that the presence of that topic positively predicted activity in a given contributing module. In comparison to the other two modules, the DMN module was more associated with the psychological functions of mentalizing, awareness, memory, and emotion, the CEN module with inhibition and conflict, and the APN module with action, visual‐motion, gaze, and motor.

## 4 DISCUSSION

We applied in this study a prediction framework via machine learning in two independent samples of healthy participants to examine the relationship between individual differences in trust propensity (as measured by two different types of trust games) and task‐independent, multimodal brain measures (as collected from sMRI and rs‐fMRI). First, our multivariate prediction analyses revealed that individual differences in trust propensity for the first sample playing the standard trust game were predicted by gray matter volume and node strength across multiple regions (i.e., internal validation). Second, the gray matter volume of these regions further enabled the classification of individuals from an independent sample with the propensity to trust or distrust as measured with the binary trust game (i.e., external validation). Finally, our modular and functional decoding analyses revealed that the predicted regions were parts of three identified brain modules, of which the psychological functions have been previously associated with domain‐general large‐scale brain networks: DMN, CEN, and APN.

Our findings fit well with a recently proposed neuropsychoeconomic model of trust—assuming that trust is rooted in the interplay of psychological components that engage in domain‐general large‐scale brain networks (Krueger & Meyer‐Lindenberg, 2019). The one‐shot trust game measuring a person's propensity to trust represents a social dilemma in which the risk of treachery contrasted with the anticipation of reward creates uncertainty. To transform the risk of treachery into positive expectations of reciprocity, the CEN implements a calculus‐based trust strategy engaging the APN to perform cost–benefit calculations while the DMN simulates the trustworthiness of the anonymous partner.

On the one hand, as hypothesized, intrinsic structural and functional features of the DMN module—DMPFC (SFG, BA 10) and temporal cortex (STG, BA 22)—predicted individual differences in trust propensity. The functional decoding analysis showed that the psychological functions of this module were more associated with mentalizing, awareness, memory, and emotion compared to the other two modules. As key nodes of the DMN module, the DMPFC and STG are consistently associated with mentalizing (i.e., theory of mind) to simulate, explain, and predict behavior of others (Frith & Frith, 1999; Krueger, Barbey, & Grafman, 2009). The DMPFC is critical not only for self‐awareness and self‐referential processing but also in forming impressions and inferencing traits of others—in both social “offline” tasks (e.g., social judgment paradigms) and economic “online” games (Frith & Frith, 2006; Ma et al., 2012; Wilson‐Mendenhall, Simmons, Martin, & Barsalou, 2013). Mentalizing and episodic memory share common regions—including the DMPFC and STG—utilized for imagining oneself in another perspective, time, or place (Van Hoeck et al., 2013). Further, mentalizing and emotional processing share similar neural regions (Hooker, Verosky, Germine, Knight, & D'Esposito, 2008). For example, reduced activities in the DMPFC and STG are observed for impaired affective mentalizing in psychotic compared to nonpsychotic individuals (Harenski et al., 2018). Further, the DMPFC is more associated with utilitarian appraisals of moral dilemmas, whereas the STG more with emotional appraisals (Hutcherson, Montaser‐Kouhsari, Woodward, & Rangel, 2015). Finally, meta‐analytic evidence revealed that the STG is involved in the execution of cognitive emotion regulation (Kohn et al., 2014; Winecoff, Labar, Madden, Cabeza, & Huettel, 2011), where top‐down connectivity from the STG controls affective valuation in the PFC and modulates emotional responses in the amygdala (Koush et al., 2019).

Our findings support previous evidence that task‐free RSFC of DMN exclusively predicts individual differences in trust propensity in a one‐round trust game employing a prediction‐analytics framework (Bellucci et al., 2019). Further, both the STG and DMPFC are involved during trust behavior. People who tend to conform to others' opinions and behaviors (i.e., social influence) show decreased STG activity when trusting another person (Wei, Zhao, & Zheng, 2019). The gray matter volume of DMPFC is linked with individual differences in self‐reported trust propensity (Haas et al., 2015). DMPFC activity is observed during attributing and inferring traits of others to evaluate a partner's trustworthiness—not only based on prior information about the partner but also through iterative interactions with the partner (Fouragnan et al., 2013; McCabe, Houser, Ryan, Smith, & Trouard, 2001). DMPFC activity reflects whether partners progress from a calculus‐based relationship to advanced forms of trust relationships (i.e., knowledge‐based, identification‐based trust) (Krueger et al., 2007). Finally, DMPFC activity has been shown in novice chess players who employ an iterative thinking pattern about potential intentional choices of an opponent alongside chess rule‐based decision‐making (Powell, Grossi, Corcoran, Gobet, & Garcia‐Finana, 2017). Based on previous evidence, we argue that intrinsic structural and functional features of the DMN module predicted individual differences in trust propensity since this module helps simulate an anonymous partner's trustworthiness based on the implementation of a calculus‐based trust strategy.

On the other hand, as predicted, intrinsic structural and functional features of the CEN module—the LPFC (VLPFC, BA 45, 46; DLPFC, MFG, BA 9; PrCG, BA 9) and PPC (SMG, BA 40; PreC, BA 29)—predicted individual differences in trust propensity. In comparison to the other two modules, the psychological functions of the CEN module were more associated with conflict and inhibition. The cognitive control system is anchored in the LPFC (i.e., DLPFC, VLPFC) within the CEN, which has been consistently associated with high‐level cognitive functions (e.g., inhibition, conflict resolution) in regulating, integrating, and adopting goal‐directed behaviors under changing context (Miller & Cohen, 2001). Both the DLPFC and VLPFC are activated during trust decisions. For example, the DLPFC responds differently when learning to trust individualistic compared to cooperative counterparts (Lemmers‐Jansen, Krabbendam, Veltman, & Fett, 2017). In general, the DLPFC provides the cognitive capacity for resolving conflict, as seen in social dilemmas such as trust—eliminating the uncertainty between the risk of treachery and the anticipation of reward (Krueger & Meyer‐Lindenberg, 2019). Further, the VLPFC disrupts the impact on learning (via the dorsal STR) after violations of trust when priors about the trustee are present—maintaining choices anchored with reliable prior beliefs (Fouragnan et al., 2013). Overall, the VLPFC grants the cognitive capacity for inhibiting information about social risk to maintain a positively biased expectation of a partner's reciprocity (Krueger & Meyer‐Lindenberg, 2019). Based on previous evidence, we argue that intrinsic structural and functional features of the CEN module predicted individual differences in trust propensity since this module likely provides the cognitive capacities of resolving the conflict of uncertainty and inhibiting information about the risk of treachery to transform it into a positive expectation of reciprocity.

Further, we argue that the posterior parietal regions (SMG, BA 40; PreC, BA 29) of the CEN module in conjunction with the pre‐motor (PrCG, BA 6) and primary motor (PoCG, BA 4), posterior parietal (SPL, BA 7), and occipital (MOG, BA 18) regions of the APN module enabled cost–benefit calculations. The APN module was linked with the psychological functions of action, motor, gaze, and visual‐motor compared to the other two modules. The embodied cognition framework suggests that neural systems for both action and perception are engaged in higher cognitive processes (Dehaene & Cohen, 2007; Tschentscher, Hauk, Fischer, & Pulvermuller, 2012). For example, developmental studies show a link between numbers and individual finger counting movements due to the acquisition of numerical skills through finger counting while counting objects and solving simple counting problems in childhood (Butterworth, 1999; Lindemann, Alipour, & Fischer, 2011). Those systematic sensory‐motor neural activities during number acquisition remain part of the numerical knowledge in our later life (Lakoff & Núñez, 2000).

A plethora of studies have confirmed this anatomical overlap of neuronal activity for numerical processing and performance in simple arithmetic tasks in addition to grasping movements and pointing (Pesenti, Thioux, Seron, & De Volder, 2000; Zago et al., 2001)—driven by parietal cortical areas (e.g., SPL, SMG, PreC) subsequently activating pre‐motor (e.g., PrCG) and primary motor (e.g., PoCG) areas eliciting the sub‐threshold tendency to move associated fingers (Butterworth, 1999; Rusconi, Walsh, & Butterworth, 2005). Moreover, neuroimaging findings in adults suggest specific number and generalized magnitude processing as well as exact and approximate number processing rely on distinct neural circuits. For example, a recent neuroimaging meta‐analysis revealed specific SPL activity for symbolic numerical magnitudes (i.e., Arabic digits and number words) but specific PreC activity for non‐numerical magnitudes (e.g., physical size, duration, or luminance) (Sokolowski, Fias, Bosah Ononye, & Ansari, 2017). Further, arithmetic calculations in symbolic formats (e.g., Arabic digits) showed increased activity in SMG, whereas arithmetic in the non‐symbolic format (e.g., dot arrays) showed increased activities in SPL and MOG (Peters, Polspoel, Op de Beeck, & De Smedt, 2016). Based on previous evidence, we argue that intrinsic structural and functional features of the APN module predicted individual differences in trust propensity since this module possibly helps to perform a cost–benefit analysis in calculating how much money to send over to and how much to expect back from the other party.

In summary, we examined the prediction of individual differences in trust propensity based on multimodal, task‐independent brain measures in two independent samples completing two behavioral measures of trust propensity. Our multivariate prediction analyses revealed that individual differences in trust propensity were predicted by intrinsic structural (i.e., GMV) and functional (i.e., gFCS) features across multiple regions. The intrinsic structural features of these regions further enabled the classification of individuals from an independent sample with a propensity of trust or distrust as measured with the binary trust game. The predicted regions have been previously implicated as modules of domain‐general large‐scale brain networks, supporting psychological processes that determine an individual's trust propensity.

A couple of limitations need to be noted. First, the current study controlled for various confound variables in a large homogeneous healthy sample. However, future studies should include additional confounding variables (e.g., personality traits, clinical characteristics) with an increased sample size to fit more complex prediction models in a more heterogeneous sample. Second, future investigations have to show re‐test reliability for our findings in which the same population completes the same trust measure but at different time points—essential for characterizing trust propensity with brain functions in health and disease as the next step. Finally, although the performance of the current prediction/classification models was significantly better than the chance level, their performance was at a moderate level and could be further improved. In this regard, the current study should be considered as a proof‐of‐concept study, and future investigations should improve the accuracy of those models. For example, only voxel‐wise GMV patterns were employed in the current prediction models. Future studies might improve the performance of prediction model by including surface‐based morphometric metrics (e.g., cortical thickness) as these metrics have been associated with human social behaviors (Baumgartner et al., 2016; Baumgartner, Schiller, Hill, & Knoch, 2013). Likewise, different levels of metrics derived from functional data (e.g., nodal or network level) should be explored in future studies.

Despite these limitations, based on internal and external validation, our results identified novel and critical evidence for intrinsic structural and functional features of multiple brain modules that are predictive of trust propensity at the individual level—supporting previous evidence from both task‐based and task‐free fMRI studies investigating the neurobiological signatures of trust. Our findings deepen not only the neuropsychological understanding of individual differences in trust propensity, but also provide potential biomarkers in an fMRI‐informed science of individual differences of trust propensity in patients with neurological and psychiatric disorders.

## CONFLICT OF INTEREST

The authors are unaware of any conflicts of interest, financial or otherwise.

## AUTHOR CONTRIBUTIONS

All authors contributed to the study conception and design. Material preparation, data collection, and analysis were performed by Chunliang Feng and Zhiyuan Zhu. The first draft of the manuscript was written by Chunliang Feng, Zhiyuan Zhu, and Frank Krueger, and all authors commented on previous versions of the manuscript. All authors read and approved the final manuscript.

## CODE AVAILABILITY

The analysis code is available from the corresponding author (C.F.) upon reasonable request.

## Supporting information


**Appendix** **S1**. Supporting Information.

## Data Availability

Data and material related to this paper are available on request from corresponding author (C.F.).
