# Supplementary material for: Prediction of trust propensity from intrinsic brain morphology and functional connectome
Source: Hum Brain Mapp. 2020 Oct 1;42(1):175–91. doi: 10.1002/hbm.25215 (PMC7721234; doi:10.1002/hbm.25215)
Supplement: Supplementary file 1 — Appendix S1. Supporting Information. [file HBM-42-175-s001.docx]

**Supporting information**

**1. T1-weighted image quality control**

All T1 weighted scans were visually checked for motion-artifacts by one of author (Z.Z.) in line with previous studies ([Reuter, et al., 2015](#_ENREF_7); [Savalia, et al., 2017](#_ENREF_8)). In addition, the quality of T1-weighted images was objectively estimated with the CAT12 toolbox (http://www.neuro.uni-jena.de/cat/), allowing for evaluating essential image parameters such as noise, inhomogeneities, and image resolution. Afterwards, all quality measures were integrated into a single quality rating (QR), indicating six levels of image quality: (i) excellent (QR: 0.5~1.5), (ii) good (QR: 1.5~2.5), (iii) satisfactory (QR: 2.5~3.5), (iv) sufficient (QR: 3.5~4.5), (v) critical (QR: 4.5~5.5), and (vi) unacceptable/failed (QR: 5.5~10.5). The quality of all T1-weighted images were good or satisfactory (i.e., QR<3.5) (**Fig. S1**) and no differences in image quality between trusting and distrusting groups were observed (*t*_(84)_ = 0.72, *P* = 0.54). Moreover, the control analyses included image quality ratings as one of the covariates to further regressed out the effects of image quality on prediction performance.

**2. Predicting trust propensity from whole-brain gFCS features**

The prediction model based on whole-brain gFCS features could not predict individual trust propensity (*r* = -0.15, *P* = 0.58, *MSE* = 2.35, *P* = 0.54, permutation test).

**3. Predicting altruistic preferences in the second sample**

The trusting group exhibited higher altruistic preferences compared to the distrusting group (**Tab. S1**), which is in line with previous findings ([Van Lange, et al., 2017](#_ENREF_10)). However, the GMV-based model of trust could not predict individual altruistic preferences (*r* = -0.28, *P* = 0.98, *MSE* = 2113.1, *P* = 0.92). Further, function values derived from classification in the trust game did not significantly correlate with altruistic preferences (*r* = -0.138, *P* = 0.204). Please note that the correlation coefficients represent the association between actual and predicted altruistic preferences, rather than between altruistic preferences and neuroimaging measures. Therefore, the negative correlation coefficients indicate the failure of the prediction model ([Greene, et al., 2018](#_ENREF_5)).

**3. Preprocessing of functional data**

Neuroimaging data analyses were performed with the DPABI software package (http://rfmri.org/dpabi) ([Yan, et al., 2016](#_ENREF_12)), which is a convenient software plug-in based on SPM (<http://www.fil.ion.ucl.ac.uk/spm>). The first 10 volumes of the functional images were discarded for signal equilibrium and participants’ adaptation to scanning noise. The images were then realigned for head movement correction. One female participant was excluded from further analyses under the criteria of head motion exceeding 2.5 mm maximum translation, 2.5° rotation and mean Jenkinson type frame-wise displacement with 0.3 mm throughout the course of scans ([Power, et al., 2012](#_ENREF_6); [Yan, et al., 2013](#_ENREF_11)). Moreover, scrubbing of high motion time points identified was further carried out to remove residual motion artifact ([Power, et al., 2012](#_ENREF_9)). To normalize functional images, participants’ structural brain images were first co-registered to their own mean functional images and then subsequently segmented. The parameters derived from segmentation were used to normalize each participant’s functional images into the standard Montreal Neurological Institute space (MNI template, resampling voxel size was 3 × 3 × 3 mm^3^). Afterwards, the linear trends of time courses were removed, and a band-pass filtering (0.01-0.10 Hz) was applied to the time series of each voxel to reduce the effect of low-frequency drifts and high-frequency physiological noise ([Biswal, et al., 1995](#_ENREF_1); [Zuo, et al., 2010](#_ENREF_13)). Subsequently, four common nuisance variables were regressed out: (i) global mean signal, (ii) WM signal, (iii) cerebrospinal fluid signal ([Fox, et al., 2005](#_ENREF_3); [Snyder and Raichle, 2012](#_ENREF_9)), and (iv) movement regressors (n = 24), including autoregressive models of motion incorporating head motion parameters (n = 6) , head motion parameters one time point before ( n = 6), and corresponding squared items (n = 12) ([Friston, et al., 1996](#_ENREF_4)).

**Reference**

Biswal, B., Zerrin Yetkin, F., Haughton, V.M., Hyde, J.S. (1995) Functional connectivity in the motor cortex of resting human brain using echo‐planar mri. MAGNET RESON MED, 34:537-541.

De La Vega, A., Yarkoni, T., Wager, T.D., Banich, M.T. (2017) Large-scale meta-analysis suggests low regional modularity in lateral frontal cortex. Cerebral Cortex, 28:3414-3428.

Fox, M.D., Snyder, A.Z., Vincent, J.L., Corbetta, M., Van Essen, D.C., Raichle, M.E. (2005) The human brain is intrinsically organized into dynamic, anticorrelated functional networks. P NATL ACAD SCI USA, 102:9673-9678.

Friston, K.J., Williams, S., Howard, R., Frackowiak, R.S., Turner, R. (1996) Movement‐related effects in fMRI time‐series. MAGN RESON MED, 35:346-355.

Greene, A.S., Gao, S., Scheinost, D., Constable, R.T. (2018) Task-induced brain state manipulation improves prediction of individual traits. Nature communications, 9:1-13.

Power, J.D., Barnes, K.A., Snyder, A.Z., Schlaggar, B.L., Petersen, S.E. (2012) Spurious but systematic correlations in functional connectivity MRI networks arise from subject motion. NEUROIMAGE, 59:2142-2154.

Reuter, M., Tisdall, M.D., Qureshi, A., Buckner, R.L., van der Kouwe, A.J., Fischl, B. (2015) Head motion during MRI acquisition reduces gray matter volume and thickness estimates. Neuroimage, 107:107-115.

Savalia, N.K., Agres, P.F., Chan, M.Y., Feczko, E.J., Kennedy, K.M., Wig, G.S. (2017) Motion‐related artifacts in structural brain images revealed with independent estimates of in‐scanner head motion. Human brain mapping, 38:472-492.

Snyder, A.Z., Raichle, M.E. (2012) A brief history of the resting state: the Washington University perspective. NEUROIMAGE, 62:902-910.

Van Lange, P.A., Rockenbach, B., Yamagishi, T. (2017) Trust in social dilemmas. Oxford University Press.

Yan, C.-G., Cheung, B., Kelly, C., Colcombe, S., Craddock, R.C., Di Martino, A., Li, Q., Zuo, X.-N., Castellanos, F.X., Milham, M.P. (2013) A comprehensive assessment of regional variation in the impact of head micromovements on functional connectomics. Neuroimage, 76:183-201.

Yan, C.-G., Wang, X.-D., Zuo, X.-N., Zang, Y.-F. (2016) DPABI: data processing & analysis for (resting-state) brain imaging. NEUROINFORMATICS, 14:339-351.

Zuo, X.-N., Di Martino, A., Kelly, C., Shehzad, Z.E., Gee, D.G., Klein, D.F., Castellanos, F.X., Biswal, B.B., Milham, M.P. (2010) The oscillating brain: complex and reliable. NEUROIMAGE, 49:1432-1445.

**
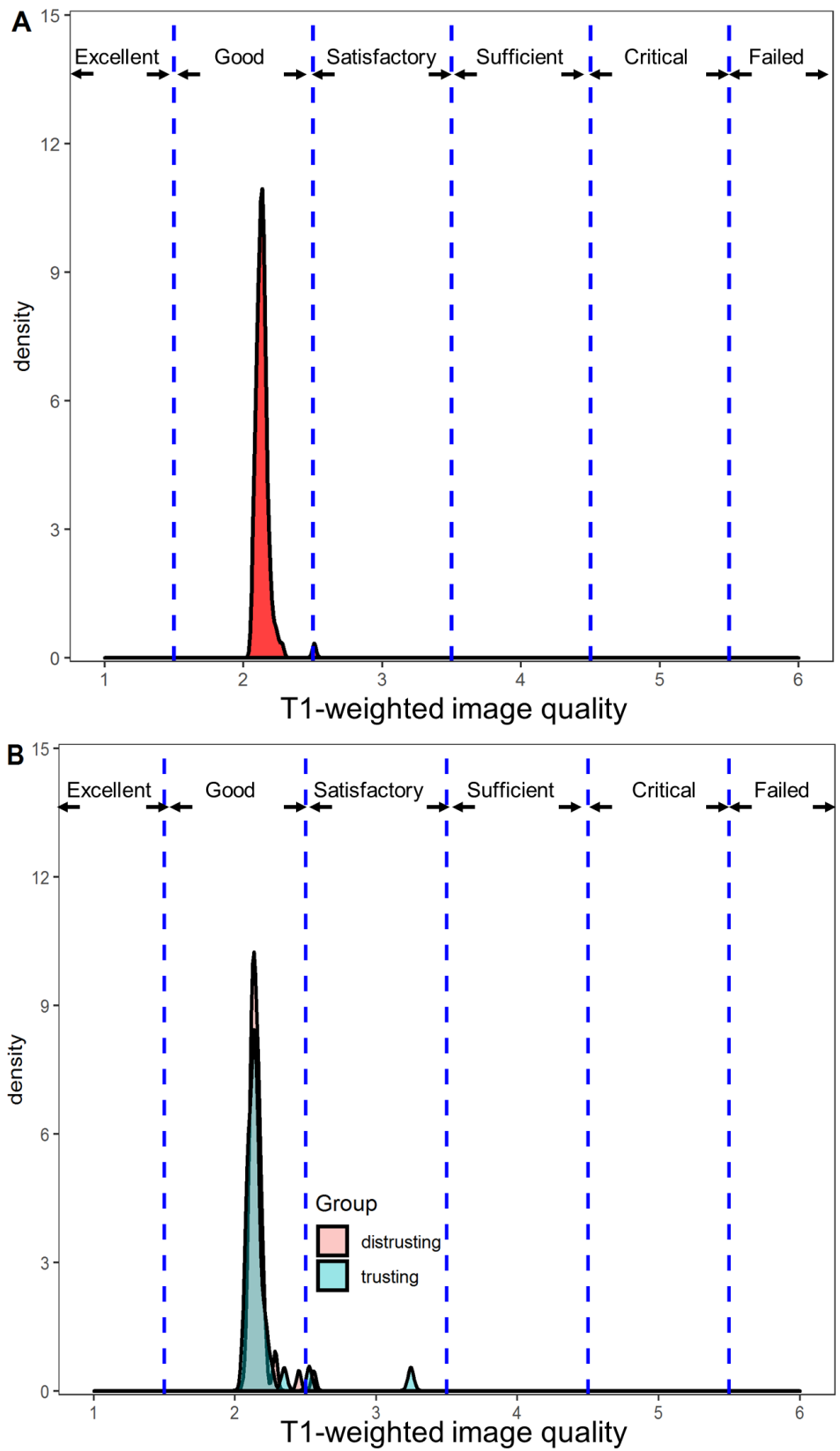
**

**Figure S1. Distribution of T1-weighted image quality rating.** **A.** Distribution of T1-weighted image quality rating of the first sample. **B.** Distribution of T1-weighted image quality rating of the second sample as a function of group (distrusting vs. trusting).

**
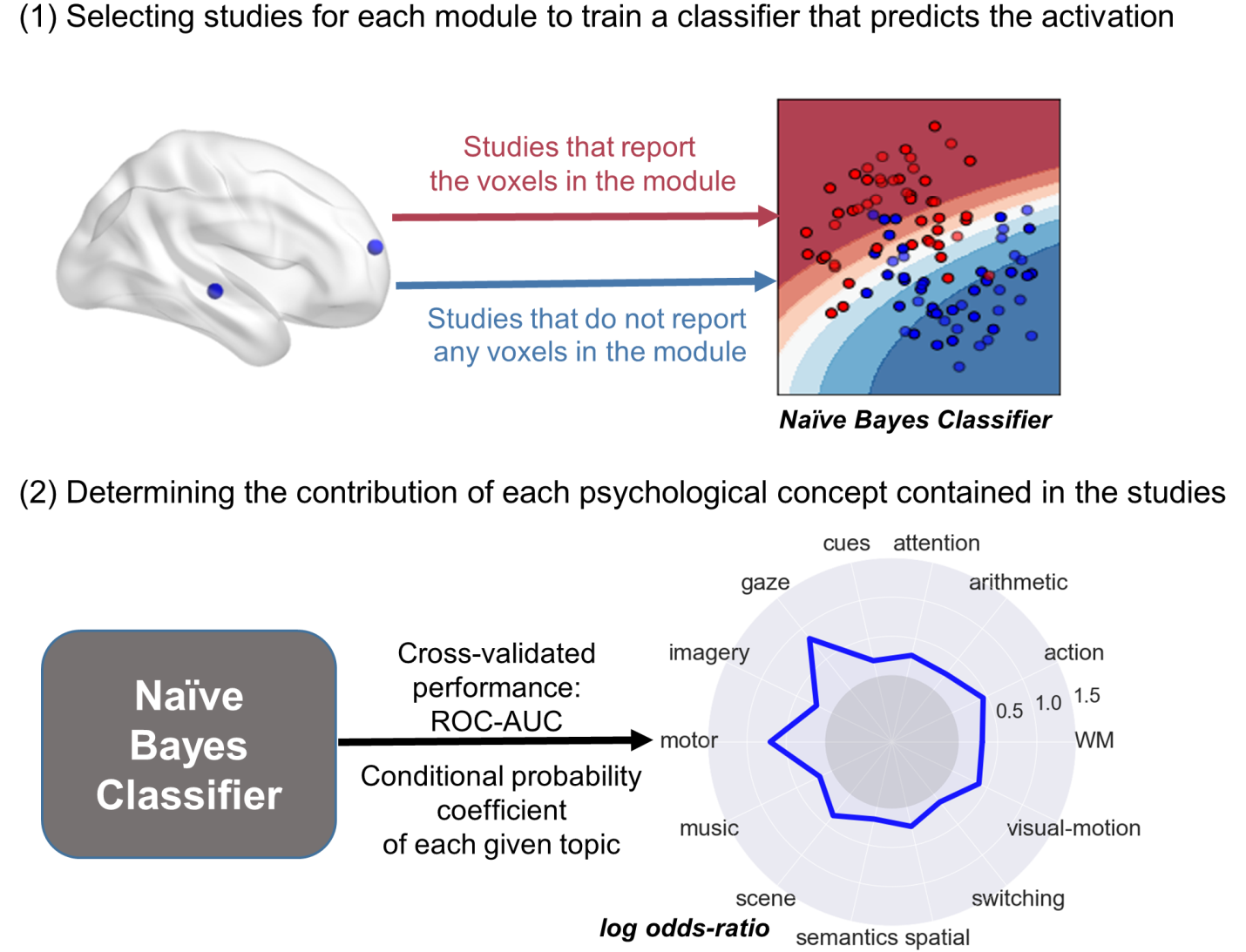
**

**Figure S2. Functional decoding framework.** Functional decoding profiles for each module were generated based on a trained machine learning model that obtained which latent psychological topics best predicted the module’s activation across studies in the Neurosynth database. The figure was inspired by a recent study ([De La Vega, et al., 2017](#_ENREF_2)).


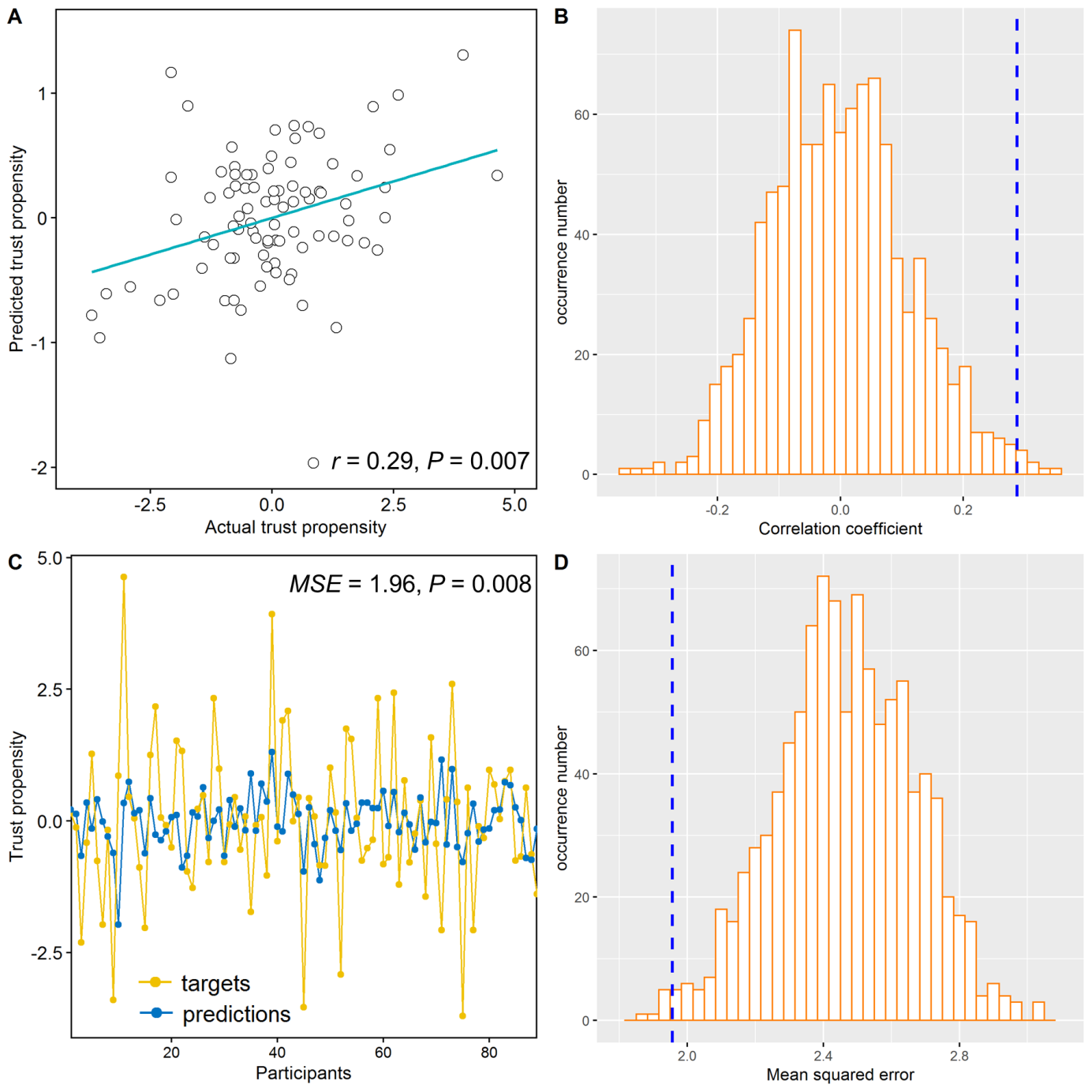


**Figure S3. Validation of the prediction model using 10-fold CV scheme.** **A.** Correlation between actual and predicted trust propensity (i.e., amounts of investment in the standard trust game). **B.** Permutation distribution of the correlation coefficient (*r*). The value obtained using real scores is indicated by the blue dashed line. **C.** Consistency between actual and predicted trust propensity. **D.** Permutation distribution of the mean squared error. The value obtained using real scores is indicated by the blue dashed line.

**Table S1. Demographics and behavioral profiles (mean and S.D.) of the participants in the external validation sample**

| **Category** | **Entire population** | **Trusting Group** | **Distrusting Group** | **Statistics** |
| --- | --- | --- | --- | --- |
| **Number (Male / Female)** | 86 (73/13) | 35 (32/3) | 51 (41/10) | χ2(1) =1.97, *P* = 0.22 |
| **Age** | 22.62 (2.37) | 22.47 (2.03) | 22.83 (2.80) | *t*(84) = -0.69, *P = 0.49* |
| **Brain size** | 1808.67 (223.47) | 1791.10 (229.05) | 1834.27 (215.78) | *t*(84) = 0.89, *P = 0.38* |
| **T1-weighted image quality** | 2.17 (0.14) | 2.19 (0.20) | 2.16 (0.08) | *t*(84) = 0.72, *P = 0.54* |
| **Altruistic preferences** | 24.33(18.89) | 30.29(17.32) | 20.24(18.99) | *t*(84) = 2.54, *P = 0.01* |

**Table S2.** Internal validation**: Contributing regions and associated weights for RSFC-based prediction model**

| Region | Hemisphere | Cluster size (voxels) | Peak MNI coordinate | | | Weights |
| --- | --- | --- | --- | --- | --- | --- |
|  |  |  | x | y | z |  |
| Superior parietal lobule (SPL) | L | 7 | -22 | -48 | 58 | 1.654 |
| Postcentral gyrus (PoCG) | L | 7 | -50 | -6 | 22 | 1.608 |
| Precentral gyrus (PrCG) | R | 9 | 46 | -6 | 32 | 1.382 |
| Superior frontal gyrus (SFG) | R | 5 | 12 | 58 | 16 | 1.357 |
| Middle frontal gyrus (MFG) | R | 7 | 26 | 32 | 32 | 1.335 |
| Precentral gyrus (PrCG) | L | 11 | -38 | 2 | 26 | 1.282 |
| Precentral gyrus (PrCG) | R | 5 | 24 | -22 | 74 | 0.99 |
| Middle occipital gyrus (MOG) | R | 12 | 24 | -84 | 14 | 0.942 |
| Superior temporal gyrus (STG) | R | 6 | 44 | -22 | -6 | 0.905 |
| Inferior fontal gyrus (IFG) | L | 13 | -34 | 36 | 10 | 0.84 |
| Supramarginal gyrus (SMG) | R | 6 | 52 | -38 | 42 | 0.766 |
| Precuneus (PreC) | R | 5 | 10 | -44 | 9 | 0.708 |
| Inferior fontal gyrus (IFG) | L | 6 | -46 | 28 | 2 | 0.222 |

R, right; L, left.

**Table S3.** External validation. Contributing regions and associated weights in the GMV-based classification model

| Region | Hemisphere | Cluster size (voxels) | Peak MNI coordinate | | | Weights |
| --- | --- | --- | --- | --- | --- | --- |
|  |  |  | x | y | z |  |
| Superior frontal gyrus (SFG) | R | 5 | 12 | 58 | 16 | 1.24 |
| Precentral gyrus (PrCG) | L | 11 | -38 | 2 | 26 | 0.93 |
| Middle frontal gyrus (MFG) | R | 7 | 26 | 32 | 32 | 0.91 |
| Precentral gyrus (PrCG) | R | 5 | 24 | -22 | 74 | 0.85 |
| Superior temporal gyrus (STG) | R | 6 | 44 | -22 | -6 | 0.79 |
| Superior parietal lobule (SPL) | L | 7 | -22 | -48 | 58 | 0.78 |
| Middle occipital gyrus (MOG) | R | 12 | 24 | -84 | 14 | 0.67 |
| Inferior fontal gyrus (IFG) | L | 6 | -46 | 28 | 2 | 0.55 |
| Precentral gyrus (PrCG) | R | 9 | 46 | -6 | 32 | 0.52 |
| Inferior fontal gyrus (IFG) | L | 13 | -34 | 36 | 10 | 0.5 |
| Precuneus (PreC) | R | 5 | 10 | -44 | 9 | 0.47 |
| Postcentral gyrus (PoCG) | L | 7 | -50 | -6 | 22 | 0.46 |
| Supramarginal gyrus (SMG) | R | 6 | 52 | -38 | 42 | 0.35 |

R, right; L, left.
